# Integrated Transcriptomic Analysis Identifies a Ferroptosis-Related Gene Signature Underlying Macrophage Heterogeneity and Immune Dysregulation in Rheumatoid Arthritis Synovium

**DOI:** 10.3390/ijms27219552

**Published:** 2026-10-26

**Authors:** Houchen Liu, Ruizhuo Zhang

**Affiliations:** 1 Faculty of Medicine, Eberhard Karls University of Tübingen, 72076 Tübingen, Germany; 2 Interfaculty Institute of Microbiology and Infection Medicine Tübingen (IMIT), Eberhard Karls University of Tübingen, 72076 Tübingen, Germany

**Keywords:** rheumatoid arthritis, ferroptosis, macrophages, single-cell RNA sequencing

## Abstract

Ferroptosis contributes to rheumatoid arthritis (RA), but the key regulators and their cell-specific functions in the synovial microenvironment remain incompletely understood. This study aimed to identify robust ferroptosis-associated biomarkers and define their cellular context through an integrated transcriptomic framework. Bulk synovial transcriptomic datasets were integrated using differential expression, weighted gene co-expression network analysis, and a curated ferroptosis gene set. Candidate genes were prioritized using three feature-selection algorithms and validated across twelve models. Immune infiltration, single-cell RNA sequencing, pseudotime, CellChat, and virtual gene perturbation analyses characterized their cellular and functional context. Six ferroptosis-related genes (*AIM2*, *HCP5*, *CAPG*, *ALOX5*, *TYMS*, and *RUNX3*) formed a diagnostic signature discriminating RA from healthy synovium (mean leakage-free test AUC 0.977 across five training-only re-derivations; 0.9375 in the primary split), with consistent leave-one-dataset-out and osteoarthritis-control validation. These genes linked to adaptive immune and ferroptosis-associated transcriptional programs. Single-cell analysis localized this transcriptional score to macrophages, where ferroptosis-signature-high macrophages occupied a central communication-network position and showed collagen-pathway enrichment; because RA and healthy samples were not fully independent of dataset origin, this finding is hypothesis-generating. Pseudotime analysis suggested progressive induction of these genes toward an inflammatory NF-κB/TNF/IL-17 terminal state, and virtual perturbation suggested convergence on mitochondrial oxidative phosphorylation networks that persisted after removing sex-chromosome genes, though residual sex-associated effects cannot be fully excluded. This study identifies a biologically interpretable ferroptosis-related gene signature and suggests ferroptosis-associated macrophage reprogramming may contribute to immune dysregulation in RA synovium. The single-cell findings are hypothesis-generating given the cohort design, while the diagnostic signature was supported by leakage-free, cross-cohort, and disease-specificity analyses, providing candidate biomarkers and therapeutic targets for RA.

## 1. Introduction

Rheumatoid arthritis is a chronic, systemic autoimmune disease affecting approximately 0.5–1% of the global population [1,2], characterized pathologically by persistent synovial inflammation, hyperplastic pannus formation, and progressive destruction of articular cartilage and bone that together drive irreversible joint damage and disability [3].

The synovial microenvironment in RA is orchestrated by a complex, self-perpetuating network of resident and infiltrating cell populations—fibroblast-like synoviocytes (FLS), macrophages, T and B lymphocytes, plasma cells, dendritic cells, and endothelial and mural cells—whose reciprocal signaling sustains chronic inflammation even when individual mediators are pharmacologically blocked [4,5]. Despite the introduction of conventional and biologic disease-modifying anti-rheumatic drugs (DMARDs), a substantial proportion of patients remain refractory to treatment [3], in part because the molecular heterogeneity of synovial pathology is incompletely resolved at single-cell resolution [6]. Clarifying the cell-type-specific programs that drive synovial inflammation therefore remains a priority for identifying more precise diagnostic biomarkers and therapeutic targets.

Ferroptosis is an iron-dependent, non-apoptotic form of regulated cell death driven by the accumulation of lipid peroxides [7], mechanistically distinguished from apoptosis, necroptosis, and pyroptosis by its dependence on labile iron, polyunsaturated fatty acid oxidation, and impaired glutathione peroxidase 4 (GPX4)–glutathione antioxidant defense [8]. Since its formal definition, ferroptosis has been implicated in the pathogenesis of a growing list of autoimmune and inflammatory conditions [9]. In RA specifically, patients display a paradoxical iron distribution—systemic iron deficiency alongside focal synovial iron overload—that promotes lipid peroxidation and ferroptotic susceptibility in synovial monocytes/macrophages, with the severity of this iron accumulation correlating with disease activity [9,10]. Ferroptotic cell death in the synovium is thought to amplify local inflammation through the release of damage-associated molecular patterns (DAMPs), which activate macrophages and other innate immune cells and trigger further pro-inflammatory cytokine release, creating a feed-forward loop between iron dysregulation, lipid peroxidation, and synovitis [11]. Ferroptosis inducers such as RSL3 have been shown to aggravate synovitis in experimental models, while several conventional and investigational anti-rheumatic agents appear to modulate ferroptotic pathways, together suggesting that ferroptosis-related genes may hold value both as mechanistic drivers of RA pathology and as candidate biomarkers of disease state [11,12].

However, most prior work linking ferroptosis to RA has relied on a single analytic layer—either bulk transcriptomic association or single-cell descriptive profiling—and has rarely combined rigorous, cross-validated feature selection with cell-resolved and spatially resolved evidence of where and in which cell states ferroptosis-related programs are active [13,14]. Bulk expression studies are confounded by the mixed cellular composition of synovial tissue, which can obscure or dilute signals originating from specific pathogenic subpopulations, whereas single-cell studies alone cannot always establish which of many differentially expressed genes have the greatest weight of independent, converging evidence (differential expression, co-expression network centrality, and gene-set-level enrichment) supporting a genuinely core regulatory role [15].

To address this knowledge gap, we developed an integrated transcriptomic framework that combines bulk transcriptomics, machine learning, and single-cell transcriptomic analyses to systematically identify ferroptosis-related regulators and define their cellular and functional roles within the RA synovial microenvironment. By integrating complementary evidence from differential expression, gene co-expression, predictive modeling, and cell-resolved analyses, this framework prioritizes robust candidate genes while linking molecular signatures to specific cellular states and intercellular communication networks.

## 2. Results

### 2.1. Differential Expression and Co-Expression Network Analysis Nominate RA-Associated Modules

**Figure 1:**
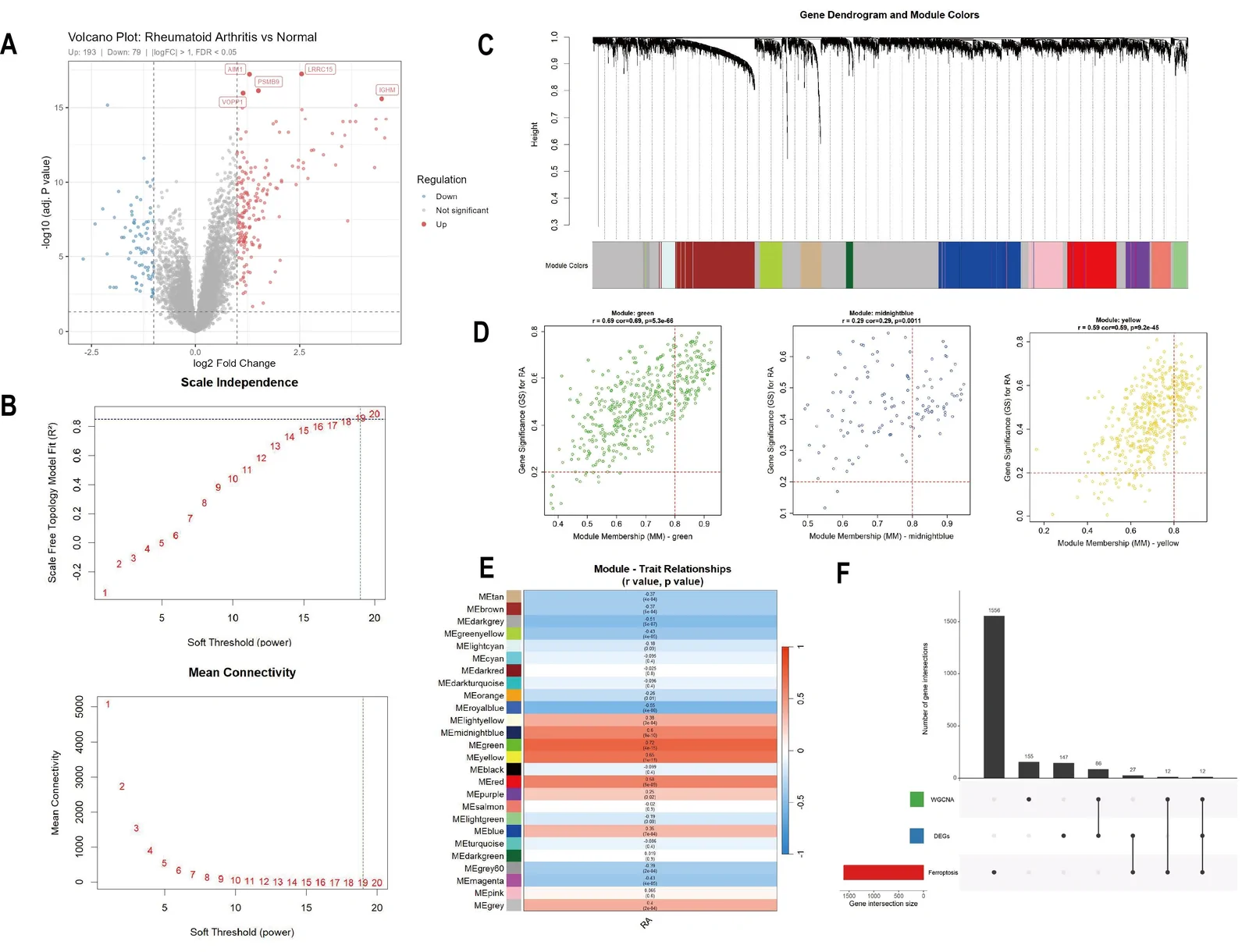
Identification of candidate ferroptosis-related genes by differential expression analysis and weighted gene co-expression network analysis (WGCNA). (**A**) Volcano plot of differentially expressed genes (DEGs) between RA and normal synovial tissue in the ComBat-corrected merged microarray dataset (193 up-regulated and 79 down-regulated genes; |log_2_ fold change| > 1, adjusted *p* < 0.05). Representative highly up-regulated genes are labeled. (**B**) Scale-free topology fit index (R^2^) and mean connectivity across soft-thresholding powers used to determine the optimal WGCNA soft-thresholding power (power = 19). (**C**) Hierarchical clustering dendrogram showing gene modules identified by dynamic tree cutting, with module assignments indicated by color. (**D**) Scatter plots of gene significance (GS) versus module membership (MM) for the green, midnight blue, and yellow modules. (**E**) Heatmap of module–trait relationships showing Pearson correlation coefficients (*r*) and corresponding *p* values between module eigengenes and RA status. (**F**) UpSet plot showing the overlap among WGCNA hub genes, DEGs, and the FerrDb ferroptosis-related gene set. Twelve genes were shared across all three datasets.*p* values are shown in exponential notation (e.g., 4e-04 = 4 × 10^−4^)

Differential expression analysis of the ComBat-corrected, merged microarray dataset identified 272 genes significantly dysregulated between RA and normal synovial tissue (193 up-regulated, 79 down-regulated; |log^2^ fold-change| > 1, adjusted *p* < 0.05). *AIM1*, *PSMB9*, *LRRC15*, *VOPP1*, and *IGHM* were among the most significantly up-regulated transcripts (Figure 1A).

WGCNA was performed on the same batch-corrected matrix at a soft-thresholding power of 19, at which the scale-free topology fit index approached its plateau while mean connectivity remained low (Figure 1B), yielding 26 co-expression modules (Figure 1C). Correlating module eigengenes with the RA disease status identified thirteen modules significantly associated with the trait (|r| > 0.3, *p* < 0.05), with the green (r = 0.72), yellow (r = 0.65), and midnight blue (r = 0.6) modules showing the strongest positive correlation with RA (Figure 1E). Gene significance for RA correlated positively with module membership within each of these three modules (green: r = 0.69; midnight blue: r = 0.29; yellow: r = 0.59), confirming that the genes most central to each module were also those most associated with disease status (Figure 1D). Intersecting the significant DEGs, WGCNA hub genes, and the FerrDb ferroptosis gene set yielded a common set of candidate ferroptosis-related genes for downstream analysis (Figure 1F).

### 2.2. Functional Enrichment Implicates Adaptive Immune and Ferroptosis-Related Pathways

**Figure 2:**
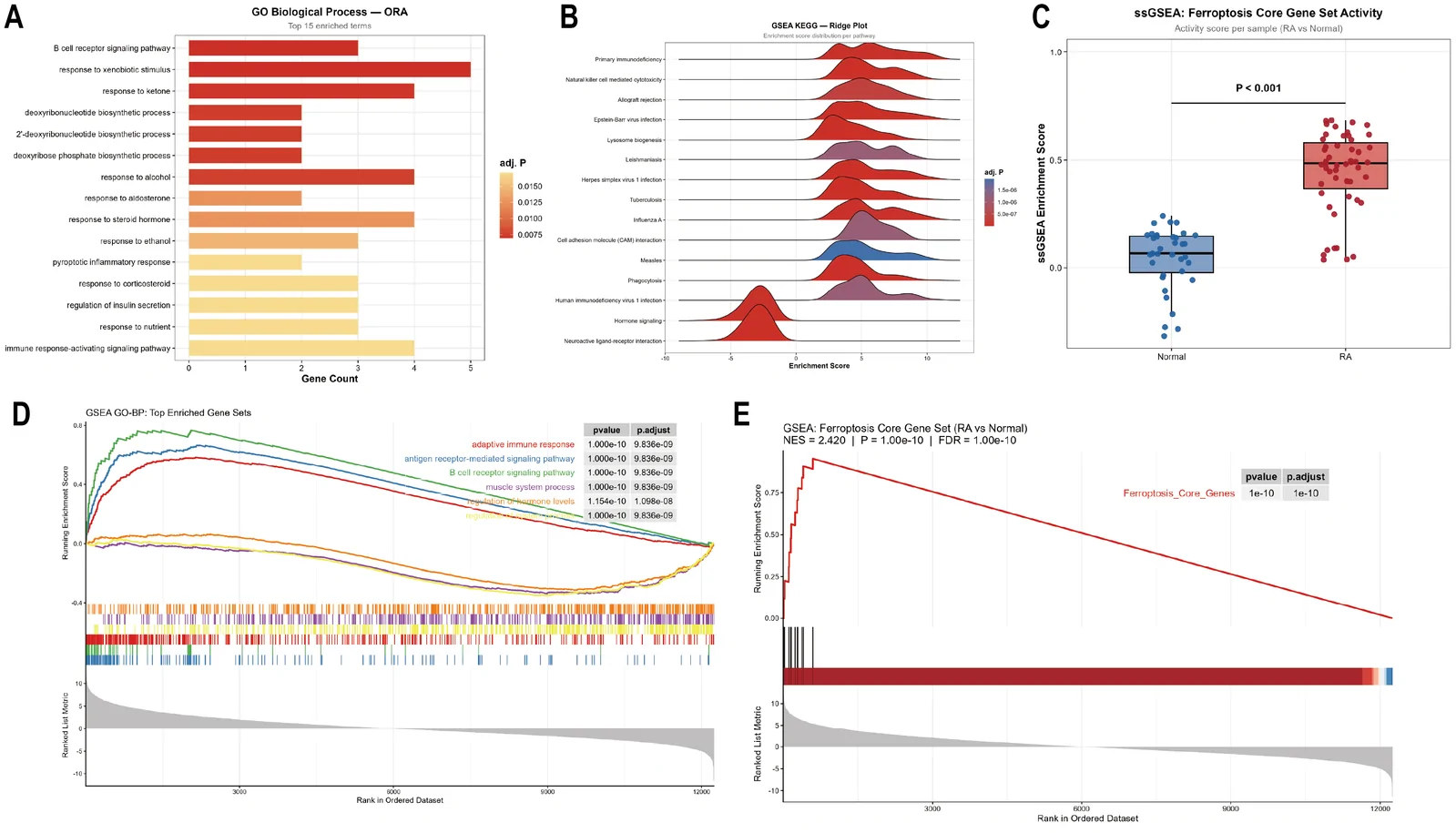
Functional enrichment analysis of candidate ferroptosis-related genes. (**A**) Top 15 Gene Ontology Biological Process (GO-BP) terms identified by over-representation analysis (ORA), ranked by gene count and colored according to Benjamini–Hochberg-adjusted *p* values. (**B**) Ridge plot showing gene set enrichment analysis (GSEA) results for the top enriched KEGG pathways, colored by adjusted *p* value. (**C**) Single-sample GSEA (ssGSEA) scores of the ferroptosis gene set in normal (*n* = 36) and RA (*n* = 51) synovial tissues (Wilcoxon rank-sum test). (**D**) Running enrichment score plots for the top-ranked GO-BP gene sets identified by GSEA, with corresponding nominal *p* values and false discovery rates (FDRs). (**E**) GSEA enrichment plot of the ferroptosis gene set.

Over-representation analysis of the intersection gene set showed enrichment for B cell receptor signaling, response to xenobiotic stimulus and ketone, deoxyribonucleotide biosynthetic process, and immune response-activating signaling pathway, among other GO Biological Process terms (Figure 2A). GSEA against the KEGG database showed positive enrichment of primary immunodeficiency, natural killer cell-mediated cytotoxicity, allograft rejection, and several viral infection-response gene sets, alongside negative enrichment of neuroactive ligand-receptor interaction and hormone signaling (Figure 2B). Consistent with this immune-skewed profile, GSEA against GO-BP gene sets showed the strongest positive enrichment for the B cell receptor signaling pathway, antigen receptor-mediated signaling pathway, and adaptive immune response (Figure 2D).

To directly test ferroptosis pathway activity, single-sample GSEA of the core ferroptosis gene set showed significantly higher enrichment scores in RA samples than in normal samples (*p* < 0.001, Figure 2C), and a dedicated GSEA confirmed strong positive enrichment of the ferroptosis core gene set in the RA transcriptome (normalized enrichment score = 2.420, *p* = 1.00 × 10^−10^, FDR = 1.00 × 10^−10^; Figure 2E). Together, these enrichment analyses indicate that the candidate gene set is embedded within both adaptive immune signaling and ferroptosis-related transcriptional programs that are coordinately activated in the RA synovium.

### 2.3. Machine Learning Identifies a Six-Gene Ferroptosis Core Signature with High Diagnostic Performance

**Figure 3:**
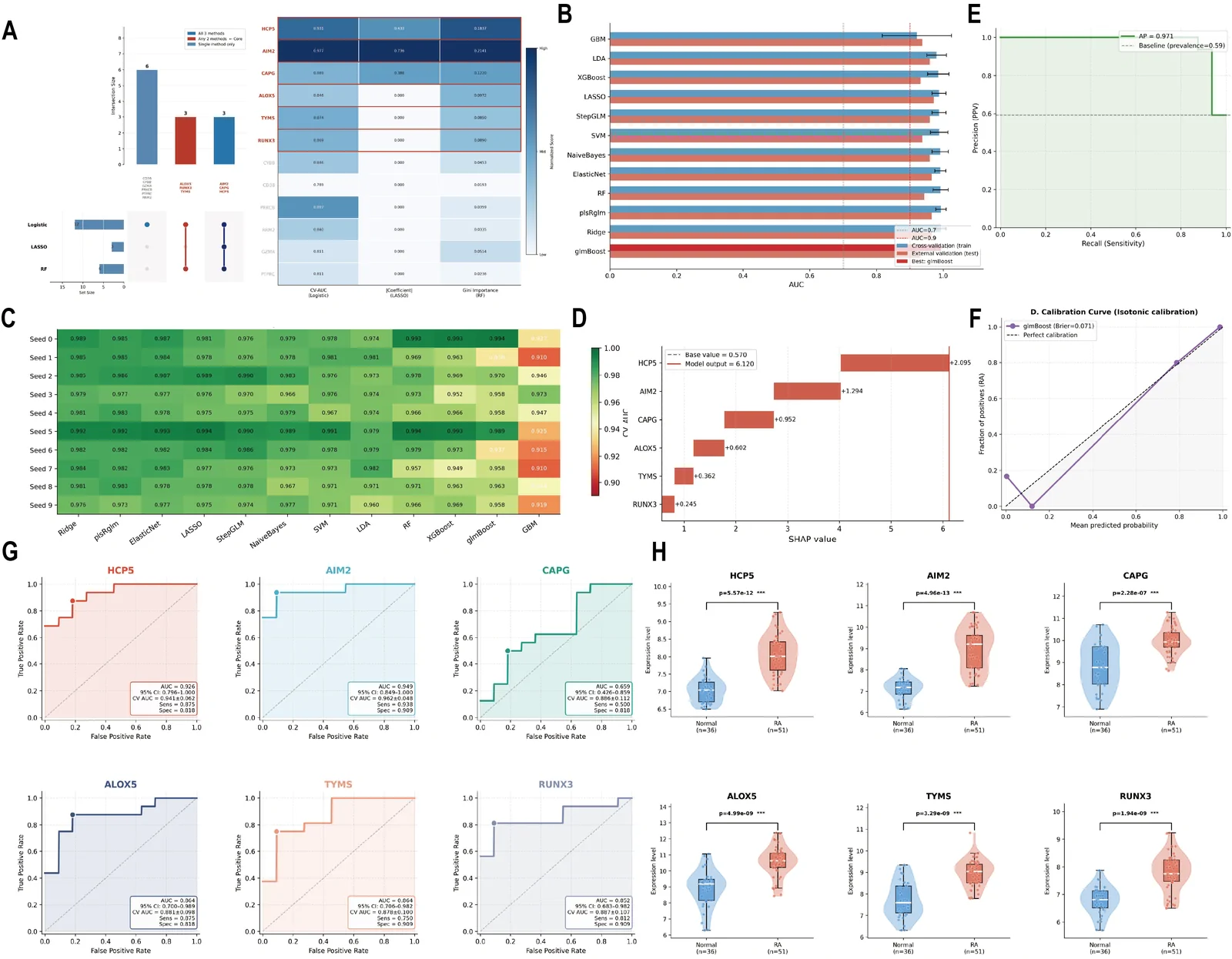
Identification and validation of a six-gene ferroptosis signature using machine learning. (**A**) Left: UpSet plot summarizing candidate genes selected by univariate logistic regression, LASSO regression, and random forest feature selection. Genes identified by at least two algorithms were retained as the core signature. Right: Heatmap showing standardized feature importance across the three feature selection methods, with the six core genes highlighted. (**B**) Cross-validated training-set and independent test-set area under the receiver operating characteristic curve (AUC) for twelve classification algorithms. (**C**) Heatmap showing cross-validated AUC values across ten independent random data partitions, illustrating model stability. (**D**) SHAP (SHapley Additive exPlanations) waterfall plot for a representative sample, showing the contribution of each core gene to the predicted outcome. (**E**) Precision–recall curve of the final glmBoost model evaluated on the independent test set. (**F**) Calibration plot of the glmBoost model generated using isotonic regression. (**G**) Receiver operating characteristic (ROC) curves of the six core genes individually, with AUCs, 95% confidence intervals, sensitivities, and specificities. (**H**) Expression of the six core genes in normal (*n* = 36) and RA (*n* = 51) synovial tissues (Wilcoxon rank-sum test).*** *p* < 0.001.

From the twelve ferroptosis-related candidate genes surviving the DEG–WGCNA–FerrDb intersection, three independent feature selection approaches—cross-validated univariate logistic regression, LASSO regression, and random forest Gini importance—were applied to the training set. Six genes (*AIM2*, *HCP5*, *CAPG*, *ALOX5*, *TYMS*, and *RUNX3*) were selected by at least two of the three methods and were designated as the core ferroptosis gene signature, while *CYBB*, *CD38*, *PRKCB*, *RRM2*, *GZMA*, and *PTPRC* were each supported by only a single method (Figure 3A). Among the core genes, *AIM2*, *HCP5*, and *CAPG* were selected by all three approaches (CV-AUC of 0.977, 0.931, and 0.869, respectively), while *ALOX5*, *TYMS*, and *RUNX3* were each supported by two of the three approaches, with zero LASSO coefficients but positive univariate significance and random forest importance.

Benchmarking twelve classification algorithms using the six-gene signature as input showed that most models achieved cross-validation and external test AUCs above 0.9, with glmBoost yielding the best overall performance and selected as the final model (Figure 3B). The final model showed high discriminative accuracy on the held-out test set, with an average precision of 0.971 against a class prevalence baseline of 0.59 (Figure 3E), and isotonic calibration analysis indicated good agreement between predicted and observed probabilities (Brier score = 0.071; Figure 3F). Model performance remained stable across ten independent random data partitions, with cross-validated AUC generally exceeding 0.95 for most algorithms other than GBM (Figure 3C).

SHAP analysis of a representative sample illustrated the relative contribution of each core gene to the model output, with *HCP5* and *AIM2* contributing the largest positive shifts from the model’s base value (Figure 3D). Consistent with this ranking, individual gene ROC analysis showed that *HCP5* and *AIM2* had the highest discriminative accuracy (AUC = 0.926 and 0.949, respectively), followed by *ALOX5* (AUC = 0.864), *TYMS* (AUC = 0.864), *RUNX3* (AUC = 0.852), and *CAPG* (AUC = 0.659) (Figure 3G). All six core genes were significantly up-regulated in RA relative to normal synovial samples (all *p* < 0.001, Wilcoxon rank-sum test), with the largest fold-differences observed for *HCP5*, *AIM2*, and *ALOX5* (Figure 3H).

#### 2.3.1. Leakage-Free Sensitivity Analysis

Because ComBat correction, differential expression analysis, and WGCNA were originally performed on the full 87-sample cohort prior to the 70/30 split, we assessed whether this could have inflated the reported diagnostic performance. Repeating the full candidate-gene derivation pipeline using only training-fold data across five independent splits, the six-gene panel achieved a mean leakage-free test AUC of 0.977 (SD = 0.021; calibrated AUC 0.979) using the glmBoost model, closely matching the AUC reported in the primary 70/30 split (raw 0.9318, isotonic-calibrated 0.9375; the residual numerical difference reflects the discrete nature of AUC at *n* = 27 test samples and minor shifts from restricting ComBat to training-fold data). At the level of individual gene selection, the training-fold-only pipeline retained *AIM2* and *ALOX5* in four of five splits, *TYMS* in three of five, *CAPG* in two of five, and *HCP5* and *RUNX3* in one of five (Supplementary Table S2). Only *AIM2* and *ALOX5* reached this ≥4/5 threshold; *TYMS*, *CAPG*, *HCP5*, and *RUNX3* showed more limited selection stability across splits and should be interpreted with corresponding caution. Despite this gene-level variability, model-level performance remained stable across all five splits (mean leakage-free test AUC 0.977), indicating that the panel’s overall diagnostic performance is not driven by any single split and is unlikely to be an artifact of leakage in the original derivation pipeline.

#### 2.3.2. Cross-Cohort and Cross-Platform Validation

Leave-one-dataset-out validation was performed across all four bulk cohorts (Supplementary Table S4). Because GSE12021 and GSE55457 originate from an overlapping patient reanalysis and are not independent of one another at the patient level, we focus the interpretation on the two patient-independent folds: leaving out GSE55235 (GPL96) yielded an AUC of 0.990, and leaving out GSE77298 (GPL570), the only GPL570-platform dataset in the cohort, yielded an AUC of 0.946, providing the most informative test of cross-platform generalization. The two patient-overlapping folds (GSE12021, GSE55457) also showed high discrimination (AUC = 1.000 for both) but are not interpreted as independent evidence of generalizability given this overlap. A naive baseline using the first two principal components of the training-fold expression matrix, fit exclusively on training data with held-out samples projected using training-derived loadings, achieved consistently lower discrimination than the six-gene panel in all four folds (baseline AUC 0.81–0.89 versus panel AUC 0.95–1.00; Supplementary Table S5), indicating the panel’s discriminative signal is not attributable to a dominant global expression axis. Because the six-gene panel itself was derived using DEG, WGCNA, and FerrDb intersection performed on the full merged cohort (Section 4.2, Section 4.3 and Section 4.4) rather than re-selected independently within each LODO fold, this analysis assesses the cross-cohort transferability of a fixed panel rather than fully independent cross-cohort feature selection; the leakage-free re-derivation in Section 2.3.1 addresses feature-selection independence within the merged cohort, while this LODO analysis addresses generalization across cohorts and platforms for that fixed panel.

#### 2.3.3. Specificity Relative to Osteoarthritis

To evaluate whether the six-gene panel is RA-specific rather than a marker of general synovial inflammation, the locked diagnostic model was applied without retraining to 20 osteoarthritis (OA) synovial samples from the same GSE55235/GSE55457 batches. The model discriminated RA test samples (*n* = 16) from OA samples with an AUC of 0.911 (95% CI 0.781–1.000), indicating that the panel is not fully explained by generic synovial inflammation, although three of 20 OA samples received RA-like prediction probabilities (>0.5), indicating imperfect specificity in a subset of cases. Psoriatic arthritis controls were not available in the datasets used in this study.

### 2.4. RA Synovium Shows a Shift Toward Macrophage and Plasma Cell Infiltration That Tracks Core Ferroptosis Gene Expression

**Figure 4:**
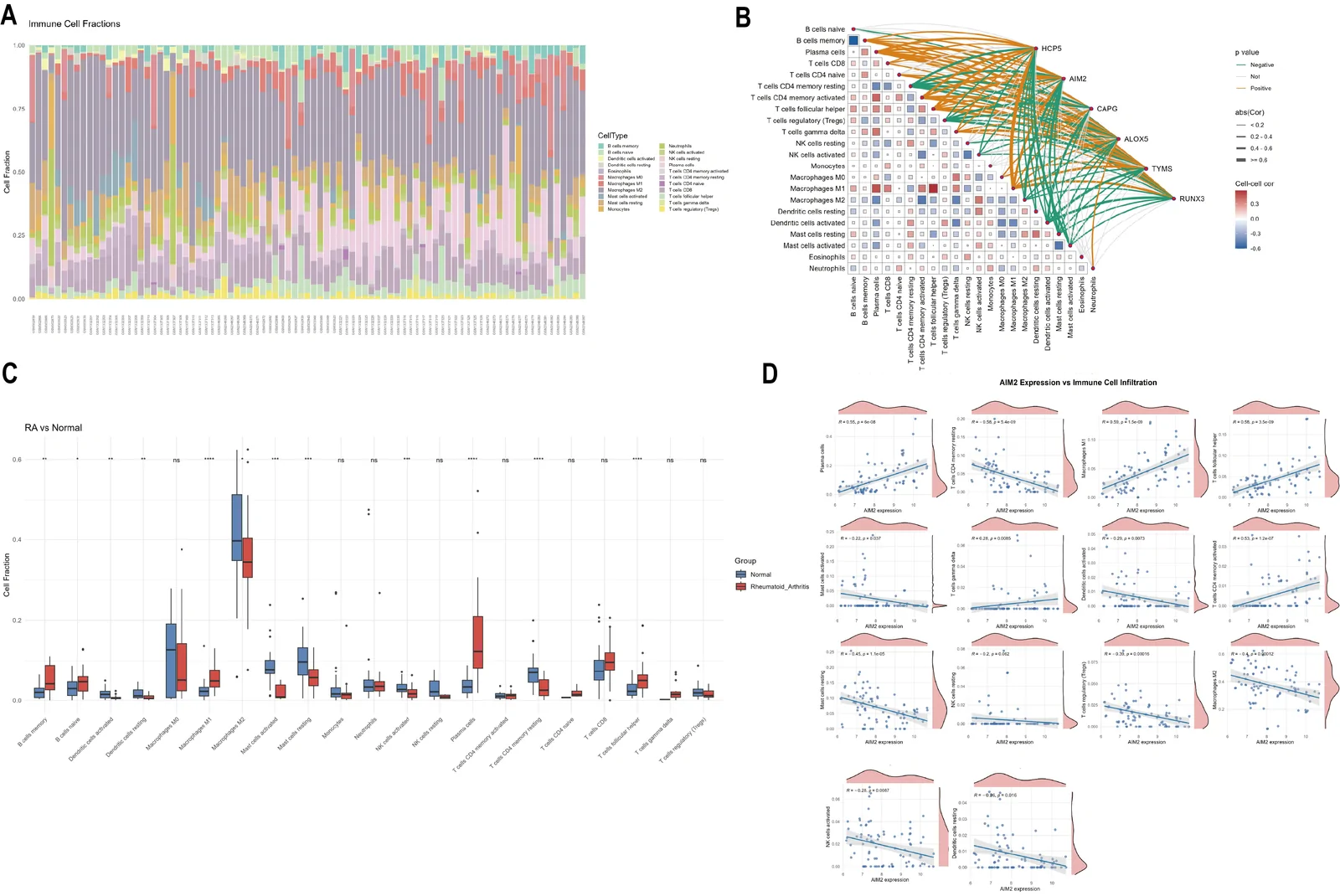
Immune cell infiltration analysis and its association with the six core ferroptosis genes. (**A**) Stacked bar plot showing the estimated proportions of 22 immune cell populations across all synovial samples (normal, *n* = 36; RA, *n* = 51), inferred using CIBERSORT based on the LM22 immune cell signature matrix. (**B**) Correlation network showing associations between the six core ferroptosis genes (*HCP5*, *AIM2*, *CAPG*, *ALOX5*, *TYMS*, and *RUNX3*) and immune cell fractions. Edge color indicates the direction of the correlation (orange, positive; green, negative), and edge width represents the absolute correlation coefficient. The inset correlation matrix shows pairwise correlations among immune cell populations, with square size and color representing correlation strength and direction. (**C**) Comparison of immune cell fractions between normal and RA synovial tissues (Wilcoxon rank-sum test; ns, not significant; * *p* < 0.05, ** *p* < 0.01, *** *p* < 0.001, **** *p* < 0.0001). (**D**) Scatter plots showing the association between AIM2 expression and immune cell fractions. Pearson correlation coefficients (*R*) and corresponding *p* values are indicated, with marginal density distributions shown along both axes.

CIBERSORT-based deconvolution of bulk expression profiles revealed distinct immune cell fraction patterns across the 87 synovial samples (Figure 4A). Compared with normal synovium, RA samples showed significantly higher fractions of memory B cells, naive B cells, macrophage M1, mast cell activated, plasma cell, and T cell follicular helper, and significantly lower fractions of macrophage M2, dendritic cells activated, dendritic cells resting and T cell CD4 memory resting (Figure 4C). The magnitude of the plasma cell increase and the macrophage M2-to-M1 shift were particularly pronounced in RA samples.

Correlation analysis among the core genes and immune cell fractions showed that *HCP5*, *AIM2*, *CAPG*, *ALOX5*, *TYMS*, and *RUNX3* were each linked to multiple immune populations through both positive and negative associations, with B cell memory and macrophage M1 emerging as major correlated nodes across several core genes (Figure 4B). Focusing on *AIM2*, expression levels correlated positively with plasma cell (R = 0.55), macrophage M1 (R = 0.59), and T cell follicular helper (R = 0.58), and negatively with T cell CD4 memory resting (R = −0.58) and macrophage M2 (R = −0.4) (all *p* < 0.05; Figure 4D). A closely parallel pattern was observed for *RUNX3*, *HCP5*, *ALOX5*, and *TYMS*, with *HCP5* showing the strongest single correlation of any core gene with macrophages M1 (R = 0.88, *p* = 3.1 × 10^−13^) (Supplementary Figure S2).

*CAPG* was a partial exception to this shared pattern, showing no significant correlation with macrophages M2 (R = 0.18, *p* = 0.15) and a more restricted overall correlation profile, with its strongest association instead being a negative correlation with the dendritic cells activated (R = −0.53, *p* = 1.0 × 10^−7^). Collectively, these results indicate that higher expression of five of the six core genes tracks a shared pro-inflammatory, macrophage M1/plasma cell-skewed immune composition in the RA synovium, whereas *CAPG* shows a narrower, macrophage-restricted correlation profile consistent with a structural rather than immune-regulatory role.

### 2.5. Single-Cell Transcriptomics Localizes the Ferroptosis-Related Transcriptional Score to a Macrophage Subpopulation

**Figure 5:**
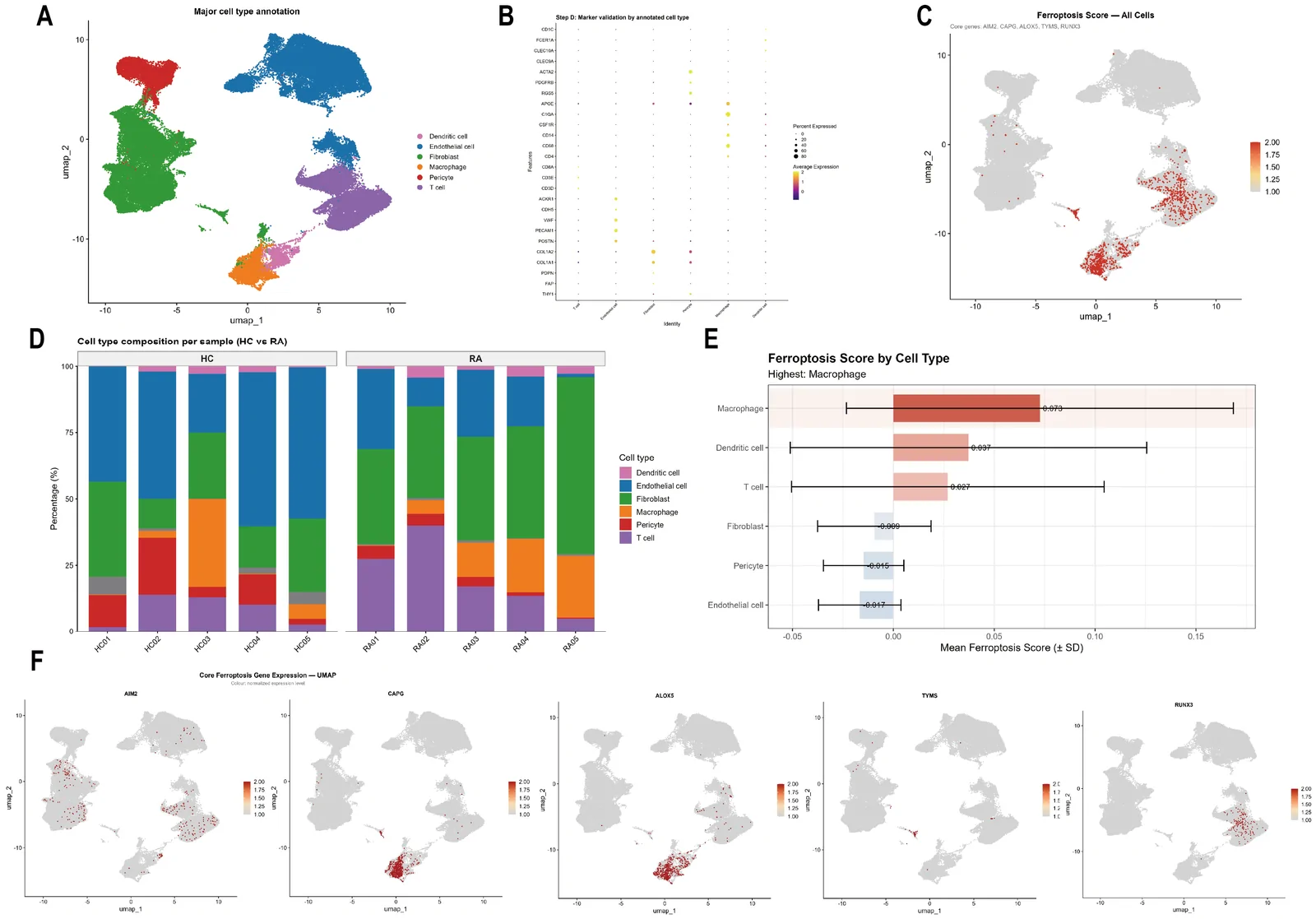
Single-cell transcriptomic characterization of the ferroptosis-related transcriptional score in RA and healthy synovium. (**A**) UMAP embedding of integrated single-cell RNA-sequencing data from healthy control (HC) and RA synovial tissues, colored by annotated major cell types. (**B**) Dot plot showing the expression of canonical marker genes used for cell type annotation. Dot size represents the percentage of cells expressing each marker, and color indicates the average scaled expression level. (**C**) UMAP embedding colored according to the ferroptosis-related transcriptional score calculated from the five detectable core ferroptosis genes (*AIM2*, *CAPG*, *ALOX5*, *TYMS*, and *RUNX3*). (**D**) Stacked bar plot showing the cellular composition of each individual sample (HC, *n* = 5; RA, *n* = 5). (**E**) Mean ferroptosis-related transcriptional scores (±SD) across annotated cell types. (**F**) UMAP plots showing the normalized expression of the five detectable core ferroptosis genes (*AIM2*, *CAPG*, *ALOX5*, *TYMS*, and *RUNX3*).

To determine which synovial cell populations express the bulk-derived ferroptosis signature, we analyzed scRNA-seq data from RA and healthy control synovium. Unsupervised clustering and marker-based annotation resolved six major cell lineages—endothelial cells, fibroblasts, pericytes, macrophages, T cells, and dendritic cells—each supported by canonical marker expression (Figure 5A,B): endothelial cells by PECAM1/VWF/ACKR1, fibroblasts by COL1A1/COL1A2, pericytes by RGS5/PDGFRB/ACTA2, macrophages by C1QA/CSF1R/CD68/CD14, T cells by CD3D/CD3E/CD4/CD8A, and dendritic cells by CD1C/FCER1A/CLEC10A.

Module scoring of the five detectable core ferroptosis genes (*AIM2*, *CAPG*, *ALOX5*, *TYMS*, *RUNX3*) showed that the ferroptosis-related transcriptional score was concentrated in the macrophage cluster and, to a lesser extent, in a subset of fibroblasts, while endothelial cells, pericytes, T cells, and dendritic cells showed largely background-level scores (Figure 5C). Quantitative scoring by cell type confirmed that macrophages had the highest mean score of all annotated lineages, followed by dendritic cells and T cells, whereas fibroblasts, pericytes, and endothelial cells showed neutral or negative mean scores (Figure 5E). This macrophage-restricted pattern was reflected at the level of individual core genes: *CAPG* and *ALOX5* showed the most prominent and cell-type-restricted expression within the macrophage cluster, *AIM2* showed broader expression across macrophages and portions of the fibroblast and T cell compartments, and *TYMS* and *RUNX3* expression was comparatively sparse (Figure 5F). Cell type composition varied considerably across individual HC and RA samples (Figure 5D). Formal comparison of sample-level cell-type proportions (Wilcoxon rank-sum test, RA *n* = 5 vs. HC *n* = 5) showed significantly higher fibroblast and lower endothelial cell proportions in RA (*p* = 0.037 for both), while the macrophage fraction, though numerically higher in most RA samples, did not reach statistical significance (*p* = 0.68) at this sample size. Leave-one-sample-out resampling confirmed that the direction of these differences was unchanged across all 10 iterations of excluding any single RA or HC sample, arguing against any one sample driving the observed pattern (Supplementary Table S3).

As in most published single-cell RA studies assembled from public repositories, RA and HC samples in this dataset originate from non-overlapping GEO series (RA: GSE200815, GSE299518; HC: GSE246416), so disease status is structurally confounded with the dataset of origin at the single-cell level. Harmony integration cannot fully separate a genuine disease effect from a systematic batch effect under this design. The composition and signaling differences reported here and in Section 2.6 should accordingly be interpreted as hypothesis-generating rather than confirmed disease effects.

### 2.6. Ferroptosis-Signature-High Macrophages Engage in Reprogrammed Collagen and SPP1 Signaling in RA

**Figure 6:**
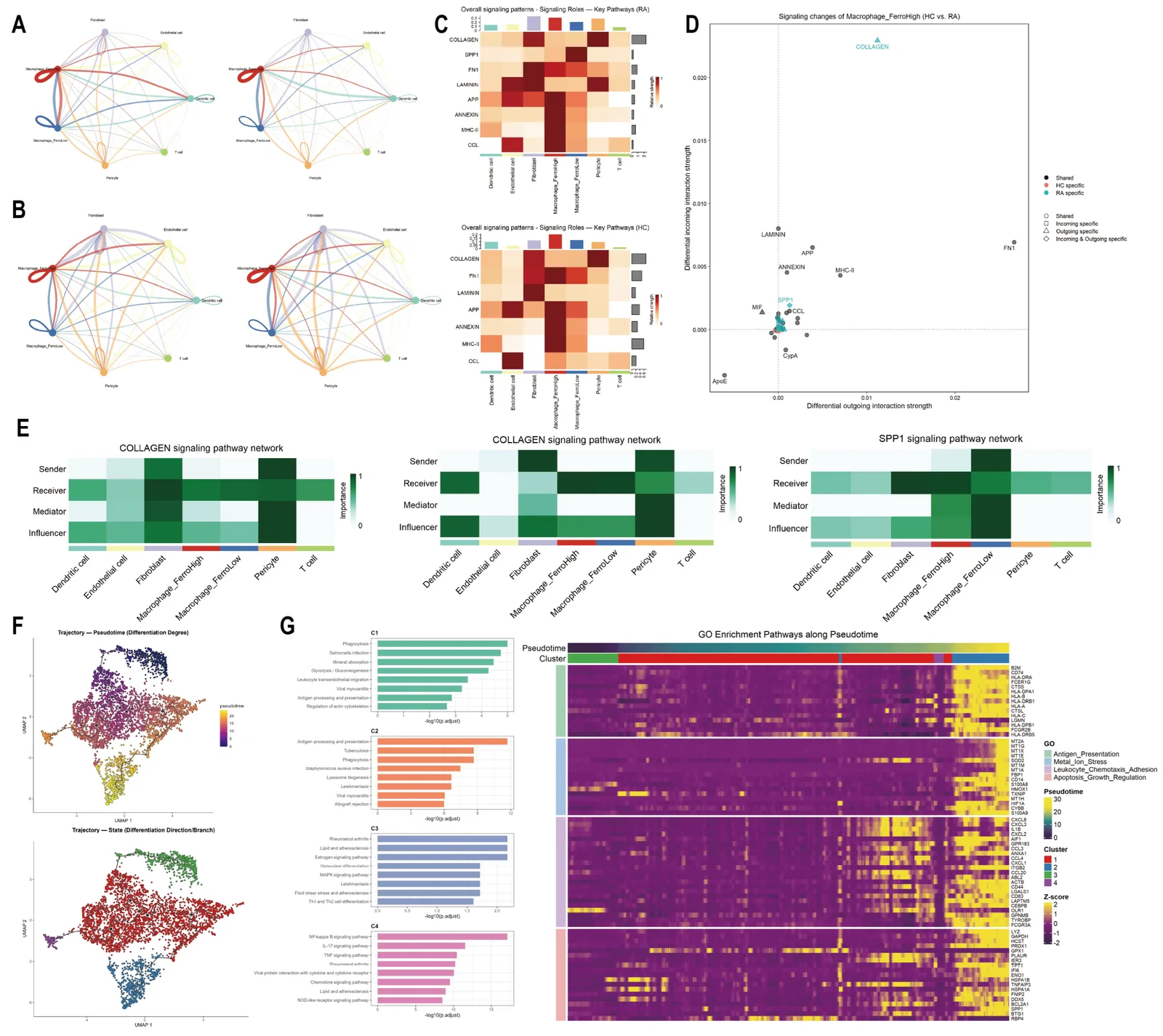
Cell–cell communication analysis and pseudotime characterization of macrophages in RA and healthy synovium. (**A**,**B**) Circle plots of CellChat-inferred intercellular communication networks among six major synovial cell populations, with macrophages further subdivided into ferroptosis-signature-high (Macrophage_FerroHigh) and ferroptosis-signature-low (Macrophage_FerroLow) subpopulations. (**A**) Healthy control (HC) synovium and (**B**) RA synovium. For each condition, the left panel depicts the number of inferred interactions and the right panel depicts the overall interaction strength. Edge width is proportional to interaction number or strength, respectively, and edge color denotes the sender cell population. (**C**) Heatmaps showing the relative signaling activity of major communication pathways (COLLAGEN, SPP1, FN1, LAMININ, APP, ANNEXIN, MHC-II, and CCL) across cell populations in RA (top) and healthy control (HC, bottom) synovial tissues. (**D**) Scatter plot comparing incoming and outgoing signaling strength of Macrophage_FerroHigh cells between RA and HC conditions. Point color indicates condition specificity (RA-specific, HC-specific, or shared), and point shape indicates the direction of specificity. (**E**) Heatmaps showing sender, receiver, mediator, and influencer signaling roles for individual cell populations in the COLLAGEN signaling pathway in RA (left) and healthy control (HC, middle) synovium, and in the SPP1 signaling pathway in RA synovium (right). (**F**) Monocle3 pseudotime trajectory of macrophages colored by pseudotime value (top) and trajectory state (bottom; states 1–4). (**G**) Left: Gene Ontology Biological Process and KEGG pathway enrichment analysis of marker genes from four pseudotime-defined clusters (C1–C4). Right: Heatmap showing scaled expression (Z-score) of representative genes along pseudotime, grouped by functional category and annotated with pseudotime values and cluster assignments.

Given the concentration of the ferroptosis-related transcriptional score within the macrophage compartment, macrophages were further stratified into ferroptosis-signature-high (Macrophage_FerroHigh) and ferroptosis-signature-low (Macrophage_FerroLow) subpopulations for cell–cell communication analysis. CellChat-inferred intercellular signaling networks showed that Macrophage_FerroHigh cells were the dominant hub of both incoming and outgoing signaling among the six major synovial cell types in both RA and healthy control tissue, with particularly strong self-signaling and reciprocal signaling with dendritic cells (Figure 6A,B).

Pathway-level signaling role analysis identified COLLAGEN, SPP1, FN1, LAMININ, APP, ANNEXIN, MHC-II, and CCL as the dominant communication pathways active in the synovial network, with Macrophage_FerroHigh cells and fibroblasts showing the strongest relative signaling strength for most pathways in both RA and healthy control conditions (Figure 6C). Comparing differential incoming and outgoing interaction strength for Macrophage_FerroHigh cells between conditions showed that COLLAGEN signaling was the pathway most distinctly enriched in the RA network relative to HC, whereas FN1, LAMININ, APP, MHC-II, and ANNEXIN were classified as shared pathways between conditions, with comparatively modest differential interaction strength relative to COLLAGEN (Figure 6D). Leave-one-sample-out sensitivity analysis showed that this COLLAGEN enrichment was preserved when any of the four GSE200815 RA samples or any HC sample was individually excluded but was undetectable when the single GSE299518 RA sample was excluded (Supplementary Figure S4C), indicating that this finding is not fully independent of that one sample and should be treated as hypothesis-generating. A within-RA comparison of the GSE299518 sample against the four GSE200815 RA samples showed it fell outside the range of the other four on most cell-type proportions and had an approximately two-fold higher macrophage ferroptosis-related transcriptional score, indicating substantial heterogeneity within the RA group itself that is not separable from the dataset of origin (Supplementary Figure S4A). Within the COLLAGEN network, fibroblasts and pericytes served as the principal signal senders and mediators, while Macrophage_FerroHigh and Macrophage_FerroLow cells were prominent signal receivers and influencers; within the SPP1 network, Macrophage_FerroLow cells occupied the dominant sender role, with fibroblasts and Macrophage_FerroHigh cells acting predominantly as receivers (Figure 6E).

### 2.7. Pseudotime Trajectory Reveals Progressive Ferroptosis Gene Induction Along Macrophage Differentiation

Pseudotime trajectory analysis of the macrophage compartment using Monocle3 resolved a differentiation trajectory spanning four transcriptionally distinct states. Pseudotime progressed from an early branch of low pseudotime value (state 3) through an intermediate-to-late transcriptional continuum occupying the bulk of the trajectory (state 1) toward two more differentiated terminal branches (states 2 and 4) (Figure 6F). Gene ontology and KEGG enrichment of cluster-specific marker genes, numbered independently along the pseudotime axis (C1–C4, not to be confused with the state labels above), showed that the earliest cluster (C1) was enriched for phagocytosis, antigen processing and presentation, and glycolysis/gluconeogenesis; C2 for antigen processing and presentation, phagocytosis, and lysosome biogenesis; C3 for rheumatoid arthritis, lipid and atherosclerosis, and MAPK signaling; and C4, corresponding to the latest pseudotime cluster, for NF-kappa B signaling, IL-17 signaling, TNF signaling, and rheumatoid arthritis pathways (Figure 6G), indicating progressive acquisition of an inflammatory, RA-relevant transcriptional program along the trajectory.

**Figure 7:**
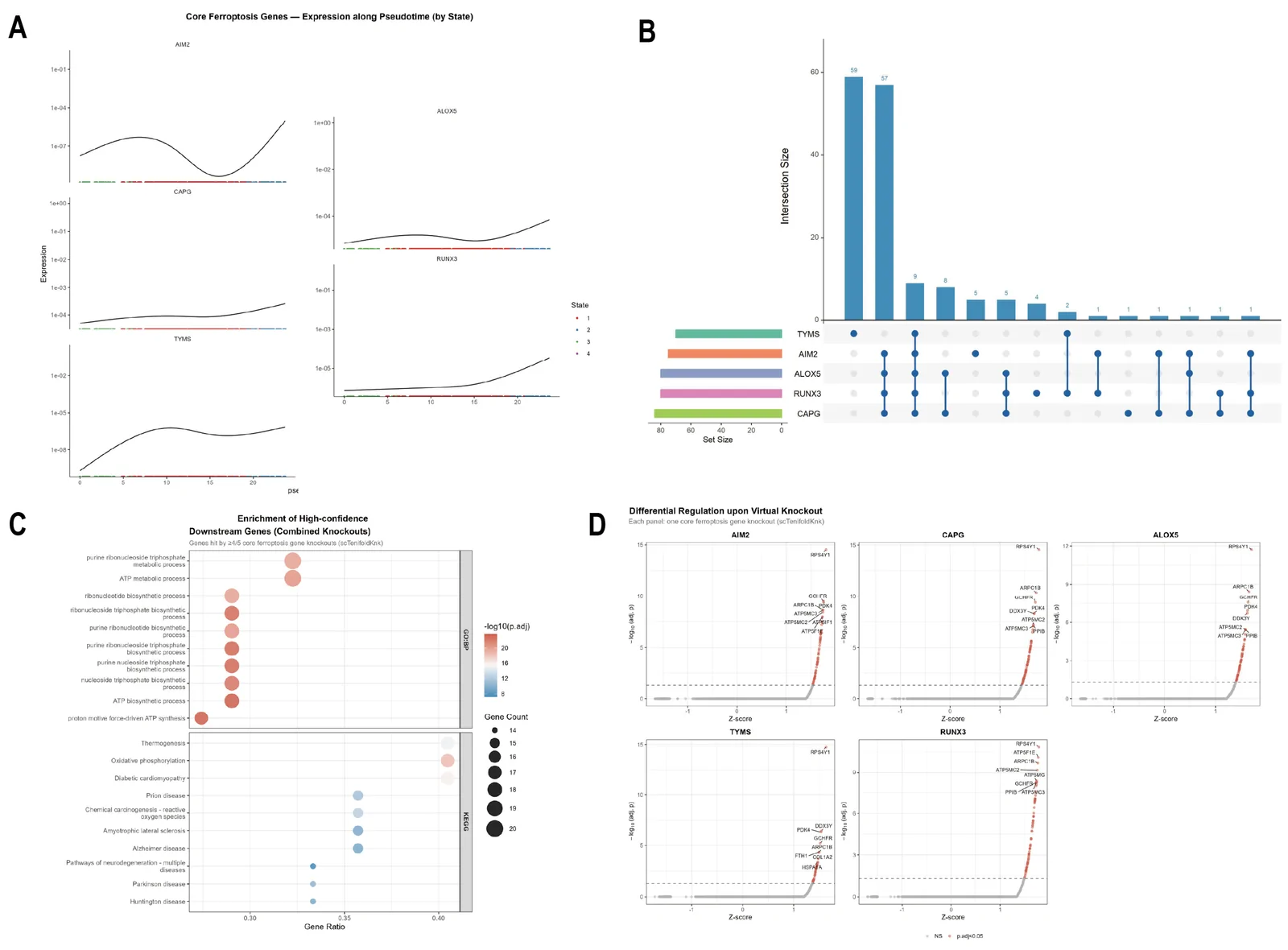
Virtual knockout analysis of the five detectable core ferroptosis genes in macrophages. (**A**) Smoothed expression trends of the five detectable core ferroptosis genes (*AIM2*, *CAPG*, *ALOX5*, *TYMS*, and *RUNX3*) along the macrophage pseudotime trajectory, colored by trajectory state. (**B**) UpSet plot showing significantly perturbed downstream genes that were unique to or shared among the five virtual gene knockout analyses performed using scTenifoldKnk. (**C**) Dot plot showing Gene Ontology Biological Process (top) and KEGG pathway (bottom) enrichment analyses of downstream genes identified in at least four of the five virtual knockout analyses. Dot size represents gene count, and color indicates −log10(adjusted *p* value). (**D**) Volcano plots showing differential regulation of downstream genes following virtual knockout of each core ferroptosis gene. The x-axis represents the Z-score, and the y-axis represents −log10(adjusted *p* value). Significantly perturbed genes (adjusted *p* < 0.05) are highlighted, and representative genes are labeled in each panel.

Expression of the core ferroptosis genes along this trajectory showed distinct but broadly convergent temporal patterns (Figure 7A). *CAPG* and *RUNX3* increased progressively with pseudotime, with the steepest rise occurring toward the terminal portion of the trajectory; *ALOX5* showed a similar overall upward trend, reaching its highest expression at the trajectory endpoint. *TYMS* expression rose earlier, peaking around mid-pseudotime before plateauing, rather than continuing to increase toward the terminal state. *AIM2* showed the most complex pattern, with an early peak followed by a decline to a trough around mid-pseudotime and a subsequent sharp rise at the trajectory endpoint. Despite this heterogeneity in timing, four of the five genes (*CAPG*, *RUNX3*, *ALOX5*, and *AIM2*) reached their highest expression at or near the terminal portion of the trajectory, positioning induction of the ferroptosis core signature as a feature of macrophages progressing toward the inflammatory, NF-κB/TNF/IL-17-enriched terminal state identified by pathway enrichment.

### 2.8. Virtual Knockout of Core Ferroptosis Genes Converges on Shared Metabolic Regulatory Programs

To explore the downstream regulatory consequences of the core ferroptosis genes within the macrophage single-cell network, virtual single-gene knockout was performed independently for *AIM2*, *CAPG*, *ALOX5*, *TYMS*, and *RUNX3* using scTenifoldKnk. The largest sets of significantly perturbed downstream genes were unique to *TYMS* (59 genes) and shared among *AIM2*, *ALOX5*, *RUNX3*, and *CAPG* (57 genes), with smaller subsets shared across other gene combinations (Figure 7B). This pattern indicates that *AIM2*, *ALOX5*, *RUNX3*, and *CAPG* converge substantially on a common downstream regulatory network, whereas *TYMS* perturbs a largely distinct, non-overlapping gene set rather than participating in this shared network.

Genes affected in at least four of the five knockouts were enriched for The ATP metabolic process, purine ribonucleoside triphosphate metabolic process, and related nucleotide biosynthetic and proton motive force-driven ATP synthesis terms among GO Biological Process categories, and for oxidative phosphorylation and thermogenesis among KEGG pathways, alongside several neurodegeneration-associated pathways (Figure 7C). Across all five individual knockout panels, *RPS4Y1*, a Y-chromosome-linked gene, consistently emerged as the most significantly perturbed downstream gene, and *ARPC1B* and *GCHFR* were recurrently identified across all five panels. Because *RPS4Y1* is Y-linked, we assessed whether this reflected donor-sex confounding rather than gene-specific regulation. Sex inference from macrophage XIST and Y-linked gene expression showed that all five HC samples were male and four of five RA samples were female (Supplementary Table S1), confounding donor sex with disease status and dataset of origin. Repeating the five virtual knockouts after removing all chromosome X- and Y-linked genes from the input eliminated *RPS4Y1* and *DDX3Y* from the downstream gene lists, consistent with these being sex-linked artifacts. The mitochondrial ATP synthase and oxidative phosphorylation signature, however, was preserved after this filtering: 68 genes remained perturbed in at least four of five knockouts, including most of the ATP synthase complex, and GO/KEGG enrichment for oxidative phosphorylation and thermogenesis was retained (Supplementary Figure S3). Components of the mitochondrial ATP synthase complex (ATP5MC2, ATP5MC3, ATP5F1E) and PDK4 were prominent downstream targets specifically within the *AIM2*, *CAPG*, *ALOX5*, and *RUNX3* knockouts, consistent with the shared 57-gene network identified in Figure 7B but were less prominent among the top TYMS-perturbed genes (Figure 7D). This convergence on mitochondrial ATP synthesis and oxidative phosphorylation-related genes across the *AIM2*, *CAPG*, *ALOX5*, and *RUNX3* knockouts suggests that these four ferroptosis-related genes are functionally linked to mitochondrial energy metabolism within RA synovial macrophages, independent of the Y-chromosome-linked genes examined here, although effects of donor sex acting through autosomal genes cannot be excluded; this relationship was not similarly supported for *TYMS* in this analysis. Because donor sex is confounded with disease status, this convergence is exploratory.

## 3. Discussion

This study combined bulk differential expression, WGCNA, and a curated ferroptosis gene set to nominate a candidate gene pool. Machine learning distilled this pool into a six-gene signature: *AIM2*, *HCP5*, *CAPG*, *ALOX5*, *TYMS*, and *RUNX3*. This signature discriminated RA from normal synovium with high accuracy. At single-cell resolution, it localized to a macrophage subpopulation central to the RA synovial signaling network. These results converge on a single working model, which should be read as hypothesis-generating: CellChat, pseudotime, and virtual knockout analyses are computational predictions rather than direct mechanistic evidence, and the single-cell composition, signaling, and perturbation findings are additionally limited by the confounding of disease status with dataset origin and donor sex, as described below. Ferroptosis-associated transcriptional reprogramming in synovial macrophages may accompany their activation and differentiation, potentially reshaping intercellular communication with fibroblasts and other stromal cells and contributing to the inflammatory circuitry of RA synovitis [10].

The six core genes were required to survive independent evidence from differential expression, WGCNA module centrality, and at least two of three orthogonal machine learning algorithms. This requirement reduces the risk of algorithm-specific artifacts. Previous bulk transcriptomic studies of ferroptosis in RA generally stopped at reporting differentially expressed genes and their pathway associations. Our pipeline carried this signature forward into single-cell space, asking where, in which cell state, and through which signaling interactions these genes act. This approach converts a statistical gene list into a cell-resolved mechanistic hypothesis.

The six core genes fall into three functional axes. These axes map onto successive stages of the model above. *AIM2* and *HCP5* form an inflammasome and innate-sensing axis, operating early in macrophage activation. *AIM2* is a cytosolic double-stranded DNA sensor that nucleates inflammasome assembly and caspase-1-dependent IL-1β release [16,17]. Elevated mitochondrial DNA in RA plasma and synovial tissue is thought to chronically engage this sensor [18]. *AIM2* has previously been reported to be increased in RA synovium and fibroblast-like synoviocytes, where its silencing suppresses synoviocyte proliferation [19]. Consistent with this, *AIM2* emerged as one of the most discriminative core genes in our model. It correlated positively with pro-inflammatory populations, including macrophages M1, plasma cells, and T cells follicular helper. It correlated negatively with macrophage M2 and resting CD4 memory T cells. This pattern positions *AIM2* as a marker of a broader pro-inflammatory state, consistent with its single-cell expression across macrophages, fibroblasts, and T cells [4]. *HCP5*, a long non-coding RNA within the extended MHC region, was similarly among the most discriminative genes in our panel. It has independently been nominated as a shared hub gene linking RA to other immune-mediated comorbidities, through proposed lncRNA and miRNA interactions [20]. Our findings reinforce this prior nomination. *HCP5* could not be reliably scored at single-cell resolution, however, owing to detection limitations inherent to droplet-based scRNA-seq. *AIM2* also needs a specific caveat. It is autosomal, but its regulation is not sex-independent. In mice, *AIM2* expression depended on cell type, was higher in male than in female splenic cells, and was increased by androgen treatment [21]. That setting is not RA synovium, so it does not establish a sex effect on *AIM2* here. It does show that an autosomal innate-sensing gene can be regulated by sex. Because donor sex is confounded with disease status in our single-cell cohort, the *AIM2* findings from these data are exploratory.

*ALOX5* anchors the ferroptosis and lipid metabolism axis, linking inflammasome priming to the lipid peroxidation machinery of ferroptosis [22]. *ALOX5* encodes 5-lipoxygenase, the rate-limiting enzyme in leukotriene biosynthesis and an established driver of ferroptotic lipid peroxidation [23]. In RA, ALOX5-derived leukotrienes trigger CD4+ T cell pyroptosis through ORAI3-dependent calcium influx. Pharmacological inhibition of *ALOX5* alleviates collagen-induced arthritis in mice, by suppressing ALOX5-dependent ferroptosis in fibroblast-like synoviocytes [24,25]. Our single-cell data extend this evidence to macrophages. *ALOX5* expression was concentrated within the macrophage cluster, closely tracking the ferroptosis module score. It also reached its highest expression toward the terminal, NF-κB, TNF, and IL-17-enriched portion of the pseudotime trajectory. This places *ALOX5* at the junction between macrophage maturation and ferroptotic lipid peroxidation, suggesting the ferroptosis-inflammation link previously shown in RA fibroblasts and T cells extends to synovial macrophages as well.

*CAPG*, *TYMS*, and *RUNX3* form a third axis, reflecting downstream cytoskeletal, metabolic, and transcriptional adaptations of an activated, differentiating macrophage. The three genes did not share a uniform temporal or network signature. *CAPG* encodes the macrophage-capping protein, with an established role in colony-stimulating factor-induced membrane ruffling and receptor-mediated phagocytosis [26]. This corresponds closely to the sharply macrophage-restricted expression of *CAPG* in our single-cell data, the most cell-type-specific of any core gene despite weak standalone discriminative power. *CAPG* most likely marks cytoskeletal remodeling during macrophage phagocytic activation, rather than acting as an independent diagnostic driver. *TYMS* encodes thymidylate synthase, the rate-limiting enzyme of de novo pyrimidine biosynthesis and the direct target of methotrexate, the anchor drug of RA treatment. Its promoter polymorphisms have repeatedly been linked to variability in methotrexate response [27]. *TYMS* rose to a mid-pseudotime peak, distinct from the terminal-state induction seen for *ALOX5*, *CAPG*, and *RUNX3*. This suggests TYMS-dependent nucleotide biosynthesis supports the proliferative demands of macrophages at an intermediate stage of activation, a previously unreported link between this pharmacogenomic target and a ferroptosis-immune axis in RA. *RUNX3* is best characterized for its roles in CD8+ T lymphocyte lineage commitment and FOXP3-dependent regulatory T cell function [28]. In our data, *RUNX3* was up-regulated in the RA synovium, detectably expressed within the macrophage cluster, and rose toward the same late-pseudotime terminal branch as *ALOX5* and *CAPG*. Upon virtual knockout, *RUNX3* converged with *AIM2*, *ALOX5*, and *CAPG* on 57 shared downstream genes, placing it within the same macrophage-intrinsic regulatory network. This is consistent with RUNX-family dysregulation having been proposed as a shared mechanistic thread across rheumatoid arthritis, psoriasis, and lupus [29].

The second stage of our model, that ferroptosis-associated reprogramming accompanies macrophage activation and differentiation, is directly supported by our pseudotime and knockout results. Trajectory analysis situated the acquisition of an NF-κB, TNF, and IL-17-enriched program at the terminal branch of macrophage differentiation, coincident with progressive induction of *ALOX5*, *CAPG*, and *RUNX3* [30] This indicates these programs are unlikely to be independently regulated. *TYMS*, by contrast, peaked earlier at an intermediate pseudotime point, suggesting its induction may precede rather than coincide with terminal activation. Virtual knockout of *AIM2*, *CAPG*, *ALOX5*, and *RUNX3* converged substantially on a shared downstream network enriched for oxidative phosphorylation and mitochondrial ATP synthesis [31]. This fits the established interdependence between mitochondrial electron transport, reactive oxygen species generation, and ferroptotic lipid peroxidation, suggesting these four genes are functionally coupled to macrophage bioenergetic state. This convergence persisted after controlling for the X/Y-linked genes examined here: repeating the analysis after excluding sex-linked genes preserved the oxidative phosphorylation signature while eliminating *RPS4Y1* and *DDX3Y* from the downstream gene lists, indicating this convergence is not driven by these Y-linked genes. Donor sex remains confounded with disease status, however. Removing sex-chromosome genes eliminates the direct contribution of X- and Y-linked transcripts. It does not remove sex-dependent regulation of autosomal genes, which is widespread across human tissues [32]. *ALOX5* shows a related pattern at the functional level: androgens lower 5-lipoxygenase-dependent leukotriene formation in human neutrophils [33]. The metabolic convergence is therefore exploratory. This design cannot show whether it reflects RA, donor sex, or both. *TYMS* knockout, however, perturbed a largely distinct, non-overlapping gene set without this metabolic signature, indicating that not all six core genes act through a single convergent mechanism [34].

The final stage of our model, that macrophage activation reshapes intercellular communication to sustain inflammation, is addressed jointly by our bulk and single-cell findings. CIBERSORT deconvolution showed the expected shift from macrophages M2 toward M1 in RA, alongside a broader remodeling of the synovial immune landscape: increased plasma cells, memory B cells, and T cells follicular helper, and reduced resting CD4 memory T cells [35]. *AIM2* expression additionally correlated negatively with Tregs at the individual-sample level, complementing the compositional shifts seen at the group level. Single-cell scoring independently identified macrophages as having by far the highest ferroptosis-related transcriptional score of any lineage, consistent with the growing literature implicating iron dysregulation and macrophage ferroptosis as active contributors to RA synovitis through DAMP release and feed-forward inflammation [10].

Beyond identifying diagnostic genes, our analysis traced their cellular origin into the intercellular signaling network. CellChat, a computational inference method, placed the ferroptosis-signature-high macrophage subpopulation at the hub of both incoming and outgoing signaling [36]. Collagen signaling showed the most distinct RA enrichment engaging this population, though as noted above this finding was not fully independent of a single cross-dataset sample and should be treated as hypothesis-generating. SPP1 signaling formed a second major axis, dominated by ferroptosis-signature-low macrophages as the sender. These findings parallel recent single-cell and spatial transcriptomic studies identifying SPP1-high macrophages near fibrin deposits and proliferating fibroblasts as drivers of hyperplastic synovial remodeling [37], and SPP1-positive macrophage-fibroblast interactions as a feature distinguishing RA from other inflammatory arthritides [4]. To our knowledge, this is the first analysis to stratify this macrophage-fibroblast axis by ferroptosis state rather than by polarization or lineage marker alone. The resulting division of labor, collagen signaling in ferroptosis-signature-high macrophages and SPP1 signaling in ferroptosis-signature-low macrophages, suggests the ferroptotic state may itself be a source of the functional heterogeneity previously observed within SPP1-associated macrophage populations, pending confirmation in a cohort where disease status and dataset origin are not collinear [38].

Our overall strategy relates conceptually to recent integrated transcriptomic investigations in other diseases, including cancer, which combine bulk transcriptomics, WGCNA, machine learning benchmarking, and single-cell CellChat analysis to define ferroptosis-related molecular subtypes [39]. Such pipelines typically build continuous prognostic risk scores for survival stratification. RA, however, is a chronic, non-malignant disease, where the pressing need is accurate discrimination between inflamed and non-inflamed synovium and identification of tractable targets. We therefore focused the machine learning stage on distilling a small, interpretable diagnostic gene set, and deliberately extended the analysis into single-cell space to ask which cell type expresses this signature and through which signaling routes it acts. Our independently derived panel converges with previously reported RA hub genes, most notably *AIM2* and *HCP5*, each separately nominated in transcriptomic analyses assembled from overlapping but non-identical GEO cohorts [19,20]. This convergence supports the robustness of our feature selection framework.

The diagnostic signature discriminated RA not only from healthy synovium but, with somewhat lower though still substantial accuracy, from osteoarthritis, arguing against the panel being a nonspecific marker of joint inflammation, though the modest OA sample size and the absence of psoriatic arthritis or other inflammatory arthritis controls limit how strongly RA specificity can be claimed.

Several identified genes also point toward therapeutic entry points. *ALOX5* is targeted by the FDA-approved inhibitor zileuton, already shown to reduce CD4+ T cell pyroptosis and joint inflammation in rodent arthritis models [24]. The macrophage expression and trajectory-linked induction of *ALOX5* observed here suggest synovial macrophages as a plausible additional cellular context for this effect. *TYMS* is the direct pharmacological target of methotrexate [27], raising the possibility that part of its anti-inflammatory action operates through the same ferroptosis-associated macrophage program identified here. The SPP1 and collagen signaling axes identified in ferroptosis-stratified macrophages align with emerging interest in macrophage and fibroblast crosstalk, rather than cytokine blockade alone, as a route to disrupting synovial hyperplasia, though this remains speculative given the single-cell cohort limitations discussed below [37].

Several limitations remain unaddressed by the analyses above. Donor age, treatment history, and other clinical variables were not available from the deposited GEO metadata and could not be incorporated into any sensitivity analysis. Psoriatic arthritis and other inflammatory arthritides were not available as controls, so full disease specificity relative to non-RA inflammatory joint disease remains unconfirmed. CellChat, Monocle3 pseudotime, and scTenifoldKnk virtual knockout analyses are computational predictions rather than direct mechanistic evidence and would benefit from future functional validation. Donor sex is the most important limitation. All five HC donors were male and four of five RA donors were female (Supplementary Table S1), so disease status in the single-cell cohort is confounded with sex as well as dataset of origin. Removing X- and Y-linked genes does not exclude sex-dependent regulation of autosomal genes. We therefore cannot determine whether the AIM2-associated findings and the metabolic convergence in the virtual knockout analyses reflect RA, donor sex, or both. These findings are exploratory and require confirmation in larger single-cell cohorts balanced or matched for donor sex.

## 4. Methods

### 4.1. Data Acquisition

Four RA synovial microarray datasets (GSE12021, GSE55235, GSE55457, and GSE77298) were downloaded from the Gene Expression Omnibus (GEO, https://www.ncbi.nlm.nih.gov/geo/, accessed on 28 April 2026) database. GSE12021, GSE55235, and GSE55457 were generated on the Affymetrix Human Genome U133A Array (GPL96), whereas GSE77298 was generated on the Affymetrix Human Genome U133 Plus 2.0 Array (GPL570). Probe IDs were annotated to gene symbols according to the corresponding platform annotation files, and multiple probes mapping to the same gene were collapsed by retaining the probe with the highest mean expression. Only genes shared across all datasets were retained for subsequent integration.

Single-cell RNA sequencing (scRNA-seq) data were obtained from three independent GEO datasets, including GSE200815 (four RA synovial samples), GSE246416 (five healthy synovial samples), and GSE299518 (one RA synovial sample), yielding a total of five RA and five healthy control synovial samples. Because all healthy control samples originated from GSE246416 and all RA samples originated from GSE200815/GSE299518, the disease status is structurally confounded with dataset of origin at the single-cell level; this design limitation and the sensitivity analyses undertaken to address it are described in Section 2.5 and the Discussion. Donor sex was inferred from macrophage XIST and Y-chromosome gene expression for each sample (Supplementary Table S1); age, treatment, and other clinical variables were not available from the deposited GEO metadata for these datasets.

A curated list of ferroptosis-related genes was retrieved from the FerrDb_V3 database (FerrDb), which catalogs experimentally validated ferroptosis drivers, suppressors, and markers.

### 4.2. Batch Correction and Differential Expression Analysis

Expression matrices from the four bulk transcriptomic datasets were merged, and batch effects were corrected using the ComBat algorithm implemented in the sva package, with dataset origin specified as the batch variable while preserving the disease status [40,41]. Batch correction performance was assessed by comparing sample-level expression distributions, principal component analysis, and hierarchical clustering of the top differentially expressed genes before and after correction (Supplementary Figure S1). Differential expression analysis between RA and healthy synovial tissues was performed using the limma package based on an empirical Bayes linear modeling framework. Genes with an absolute log2 fold change (|log2FC|) > 1 and a Benjamini–Hochberg adjusted *p* value < 0.05 were considered differentially expressed genes (DEGs). Because this batch correction, differential expression analysis, and the WGCNA and intersection steps described below (Section 4.3 and Section 4.4) were performed on the full 87-sample cohort prior to the train/test split described in Section 4.5, a leakage-free sensitivity re-analysis restricting all feature-derivation steps to the training-fold data was additionally performed, as described in Section 2.3.1 and Section 4.5.

### 4.3. Weighted Gene Co-Expression Network Analysis

Weighted gene co-expression network analysis (WGCNA) was performed using the WGCNA R package to identify gene modules associated with RA [42]. After quality control, the top 75% of most variable genes were retained for network construction. A signed weighted co-expression network was generated using a soft-thresholding power selected according to the scale-free topology criterion. Gene modules were identified using a dynamic tree-cutting algorithm with a minimum module size of 30 genes, followed by module merging at a cut height of 0.25. Module eigengenes were correlated with disease status to identify RA-associated modules. Genes with module membership (MM) > 0.8 and gene significance (GS) > 0.2 were defined as hub genes within disease-associated modules.

### 4.4. Intersection Gene Identification and Functional Enrichment

Candidate ferroptosis-related genes were identified by intersecting DEGs, WGCNA-derived hub genes, and the curated ferroptosis gene set. Functional characterization was performed using Gene Ontology Biological Process (GO-BP) over-representation analysis together with the Kyoto Encyclopedia of Genes and Genomes (KEGG) and GO-BP gene set enrichment analysis (GSEA), implemented in the clusterProfiler package [43]. In addition, single-sample gene set enrichment analysis (ssGSEA) was performed using the GSVA package to quantify ferroptosis-related pathway enrichment in each bulk sample, and pathway scores were compared between RA and healthy control groups using the Wilcoxon rank-sum test [44].

### 4.5. Machine Learning-Based Core Gene Selection

Feature selection and predictive model development were performed in Python (version 3.13.13) using the scikit-learn ecosystem [45]. To ensure reproducibility, samples were randomly divided into a training set (70%) and an independent test set (30%) by stratified sampling while preserving the class distribution. The test cohort was reserved exclusively for the final model evaluation.

To minimize method-specific selection bias, three complementary feature selection strategies were independently applied to the training cohort, including univariate logistic regression, LASSO-penalized logistic regression [46], and random forest analysis [47]. Genes consistently selected by at least two independent methods were retained as candidate core genes.

The selected genes were subsequently used to construct predictive models using twelve supervised machine learning algorithms, including penalized logistic regression, linear discriminant analysis, naïve Bayes, support vector machine, random forest, gradient boosting, XGBoost, component-wise gradient boosting, and partial least squares logistic regression. Model performance was assessed using repeated stratified five-fold cross-validation in the training cohort and independently evaluated in the held-out test cohort. Model discrimination, calibration, precision–recall performance, and stability across multiple random seeds were comprehensively assessed. Model interpretability was evaluated using SHAP analysis [48].

To assess whether candidate-gene derivation prior to the train/test split introduced information leakage, the entire feature-derivation pipeline (ComBat batch correction restricted to training-fold samples, differential expression analysis, WGCNA hub-gene identification, and FerrDb intersection) was repeated using only training-fold data, across five independent stratified 70/30 splits. For each split, the diagnostic model (glmBoost, with and without isotonic calibration) was refit exclusively on training-fold data and evaluated on the corresponding held-out test fold.

To assess robustness across cohorts and microarray platforms, leave-one-dataset-out (LODO) cross-validation was performed across the four bulk datasets. For each fold, ComBat correction was refit using only the three retained datasets as batch levels, without a disease-status covariate, and the fixed six-gene panel was evaluated using a model retrained on the retained datasets, without re-selecting features. As a check against the model exploiting a dominant global expression axis rather than the specific six-gene panel, a naive baseline using the first two principal components of the training-fold expression matrix, fit exclusively on training-fold data with held-out samples projected using training-derived loadings, was evaluated in parallel (Supplementary Table S5).

To assess whether the six-gene panel reflects RA-specific pathology rather than general synovial inflammation, the locked diagnostic model was applied, without retraining, to 20 osteoarthritis (OA) synovial samples from the GSE55235 and GSE55457 series that had not been used in any upstream analysis. Because these OA samples originate from the same batches as the primary cohort, ComBat batch-correction parameters fitted on the original 87-sample cohort were applied to the OA samples without re-fitting, and model-predicted probabilities were compared against the held-out RA test samples.

The diagnostic performance of individual core genes was evaluated using receiver operating characteristic (ROC) curve analysis, and expression differences between RA and healthy control samples were compared using the Wilcoxon rank-sum test.

### 4.6. Immune Cell Infiltration Analysis

The relative abundance of 22 immune cell populations was estimated from the batch-corrected bulk transcriptomic data using the CIBERSORT algorithm (LM22 signature matrix, 1000 permutations) implemented in the IOBR package [49,50]. Differences in immune cell infiltration between RA and healthy control samples were evaluated using the Wilcoxon rank-sum test. Associations between core gene expression and immune cell abundance were assessed using Spearman correlation analysis.

### 4.7. Single-Cell RNA-Sequencing Data Processing and Cell Type Annotation

Raw count matrices were imported into Seurat (v5) for quality control and preprocessing [51]. Genes detected in fewer than three cells and cells expressing fewer than 200 genes were initially excluded. Low-quality cells were subsequently identified independently within each sample using a median absolute deviation (MAD)-based adaptive filtering strategy implemented in the scuttle package. Cells identified as outliers in library size, detected gene number, or mitochondrial transcript proportion (>3 MADs from the median) were removed. An additional mitochondrial content threshold of 25% was applied to exclude cells with excessive mitochondrial transcript abundance. Putative doublets were identified and removed independently for each sample using scDblFinder.

Gene expression data were normalized using SCTransform (v2), followed by batch-effect correction with Harmony [52,53]. Cells were clustered on the integrated low-dimensional embedding and visualized using Uniform Manifold Approximation and Projection (UMAP).

Cell identities were determined using a consensus annotation strategy that integrated reference-based annotation with SingleR [54], large language model-assisted annotation using CASSIA [55], and manual verification based on established canonical marker genes. Major synovial cell populations included T cells, endothelial cells, fibroblasts, macrophages, pericytes, and dendritic cells.

### 4.8. Ferroptosis Scoring

A ferroptosis-related transcriptional score was calculated for each cell using Seurat’s AddModuleScore function based on the expressed core ferroptosis-related genes (*AIM2*, *CAPG*, *ALOX5*, *TYMS*, *RUNX3*). Because only *ALOX5* has an established direct mechanistic link to ferroptotic lipid peroxidation, this composite score reflects expression of a ferroptosis-associated gene program rather than direct evidence of ferroptotic cell death. Scores were compared across annotated cell populations and individual samples. Macrophages were subsequently stratified into ferroptosis-signature-high (Macrophage_FerroHigh) and ferroptosis-signature-low (Macrophage_FerroLow) subpopulations by median split of this score within the macrophage population, for downstream analyses.

### 4.9. Cell–Cell Communication Analysis

Cell–cell communication networks were inferred using CellChat with the human CellChatDB ligand–receptor database [36]. CellChat objects were constructed separately for RA and healthy controls and subsequently integrated for comparative analysis. Communication probabilities were calculated using the triMean method with population size normalization. Cell populations containing fewer than 10 cells were excluded before inference. Differential communication patterns were evaluated by comparing pathway-specific incoming and outgoing interaction strengths. Signaling roles of cell populations, including sender, receiver, mediator, and influencer functions, were characterized for representative signaling pathways. Macrophage_FerroHigh and Macrophage_FerroLow subpopulations were defined according to the ferroptosis-related transcriptional score.

### 4.10. Pseudotime Trajectory Analysis

Macrophage developmental trajectories were reconstructed using Monocle3 based on Harmony-corrected low-dimensional embeddings transferred from Seurat [56]. Principal graphs were learned using the learn_graph function, and pseudotime values were assigned using order_cells. The root state was defined according to macrophage differentiation-associated marker expression. Genes displaying significant dynamic changes along pseudotime were identified using Moran’s I statistics implemented in graph_test, followed by FDR correction. Genes significantly associated with trajectory progression were subjected to GO and KEGG enrichment analysis using expressed macrophage genes as the background set.

### 4.11. Virtual Gene Perturbation Analysis

In silico perturbation analysis was performed using scTenifoldKnk to evaluate the regulatory consequences of individual ferroptosis-related gene perturbations [57]. scTenifoldKnk reconstructs gene regulatory networks before and after simulated gene knockout and identifies differentially regulated downstream genes. Normalized macrophage expression matrices were used as input. Genes consistently affected in at least four of the five perturbation models were considered high-confidence ferroptosis-associated targets and subjected to GO and KEGG enrichment analysis.

Because *RPS4Y1* is Y-chromosome-linked, a sensitivity re-analysis was performed to distinguish donor-sex artifacts from genuine regulatory effects. All chromosome X- and Y-linked genes were removed from the input gene set prior to repeating the five virtual knockouts with identical parameters, and the resulting high-confidence downstream gene set and pathway enrichment were compared with the original analysis (Supplementary Figure S3).

### 4.12. Statistical Analysis

Statistical analyses were performed using R (4.4.3) and Python (3.13.13). Continuous variables were compared using two-sided Wilcoxon rank-sum tests unless otherwise specified. Correlation analyses were performed using appropriate correlation methods according to data distribution. Multiple testing was corrected using the Benjamini–Hochberg method. A false discovery rate (FDR) or adjusted *p* value < 0.05 was considered statistically significant. For diagnostic AUC comparisons in the cross-cohort and disease-specificity validation analyses, 95% confidence intervals were estimated using 2000 bootstrap resamples.

## 5. Conclusions

In summary, within the limits of the single-cell cohort design and the computational-prediction nature of the CellChat, pseudotime, and knockout analyses discussed above, our findings support a model in which ferroptosis-associated transcriptional reprogramming accompanies macrophage activation in RA synovium, linking innate immune sensing, lipid metabolism, and cytoskeletal adaptation to downstream macrophage-fibroblast signaling. The six-gene diagnostic signature itself was supported by leakage-free, cross-cohort, and disease-specificity analyses. The single-cell findings require confirmation in larger cohorts balanced or matched for donor sex. ALOX5 and TYMS emerge as points where existing pharmacology may already intersect with this axis, offering testable directions for future work.

## Data Availability

All datasets used in this study are publicly available in the GEO database. The analysis code supporting this study is available at Zenodo (https://doi.org/10.5281/zenodo.21923896). Further information is available from the corresponding author upon reasonable request.

## Supplementary Materials

The following supporting information can be downloaded at: https://www.mdpi.com/article/10.3390/ijms27219552/s1.

## Abbreviations

The following abbreviations are used in this manuscript:

RA	Rheumatoid Arthritis
HC	Healthy Control
WGCNA	Weighted Gene Co-expression Network Analysis
AUC	Area Under the Curve
GO	Gene Ontology
KEGG	Kyoto Encyclopedia of Genes and Genomes
GSEA	Gene Set Enrichment Analysis
GEO	Gene Expression Omnibus
DEGs	Differentially Expressed Genes
FDR	False Discovery Rate
UMAP	Uniform Manifold Approximation and Projection
ROC	Receiver Operating Characteristic
scRNA-seq	Single-Cell RNA Sequencing
